# Blood donation practice and associated factors among health professionals in Tigray regional state public hospitals, northern Ethiopia

**DOI:** 10.1186/s13104-018-3786-7

**Published:** 2018-09-24

**Authors:** Tsige Tadesse, Tadis Berhane, Teklehaymanot Huluf Abraha, Berihu Gidey, Elsa Hagos, Teklit Grum, Hadgu Gerensea

**Affiliations:** 1 Aksum Blood Bank Branch Office, Tigray Regional Health Bureau, Aksum, Ethiopia; 2 Department of Reproductive Health, School of Public Health, College of Health Sciences, Aksum University, Aksum, Ethiopia; 3 Department of Human Nutrition, School of Public Health, College of Health Sciences, Aksum University, Aksum, Ethiopia; 4 Departments of Medical Laboratory Technology, College of Health Sciences, Aksum University, Aksum, Ethiopia; 5 Department of Neonatal Nursing, School of Nursing, College of Health Sciences, Aksum University, Po.Box 298, Aksum, Ethiopia

**Keywords:** Attitude, Blood donation

## Abstract

**Objective:**

The demand for blood and blood products are increasing in all part of the globe, especially in the developing nations. However, there is limited information on the level of blood donation practice and their related factors. Therefore, assessing the level of blood donation practice and its determinant factors among health professionals have a paramount importance in designing an effective strategy for sustaining adequate and safe blood provision in the hospitals.

**Results:**

Out of 556 health professionals, 266 (47.8%) had ever donated blood in their life time. Age above 30 years (AOR = 2.756 95% CI 1.055–7.197), married health professionals (AOR = 1.729 95% CI 1.091–2.739), health professionals’ knowledge of blood donation (AOR = 3.403 95% CI 2.296–5.044), health professionals’ attitude towards blood donation (AOR = 3.41 95% CI 2.320–5.041) and health professionals who attend degree education (AOR = 0.315 95% CI 0.104–0.950**)** were significantly associated with blood donation behavior of health professionals. The magnitude of blood donation practice was found low. Therefore, the Ethiopian Red Cross Society and ministry of health should continue increasing the attitude and knowledge of health professionals toward blood donation practices are the key avenues interventions.

## Introduction

Contemporary blood transfusion is an extremely desirable component of health care delivery system. Each year millions of lives are saved through safe blood transfusion. The demand for blood and blood products are increasing in all part of the globe [1–4].

Practice towards blood donation among health professional varies in Sub-Saharan Countries, which is, 22.1% in Nigeria [5], 32.2–38.3% in Ethiopia [6–8]. As research evidence show that; gender, age, marital status, department, work experiences significantly associated with Knowledge, attitude and practice [6, 8, 9]. knowledge of the of health professionals about blood donation, the presences of family members or relatives who received blood are also the key predictors for practice toward blood donation among health professionals [2, 6]. Similarly, health professionals’ attitude towards blood donation was significantly and independently associated with blood donation behavior of health professionals [6].

In Ethiopia, blood services have been mainly provided by Ethiopian Red Cross Society (ERCS) since 1969. The main objective of then one profit society is blood bank and transfusion services include collection, processing, storage and provide human blood intended for transfusion. Blood from ERCS covered only for the need of 52% of national hospitals in the country [10–14].

Health professionals are expected to have adequate knowledge, attitude and should practiced voluntary blood donation in order to filled the scarcity of blood availability in the health facilities. However, the level of practice among the health professionals to ward blood donation is poorly assessed in the study area. Therefore, assessing the level of practice towards blood donation and its determinants factors among health professionals have a paramount in designing an effective strategy for sustaining adequate and safe blood provision in the hospitals.

## Main text

### Methods

#### Study design, period and setting

The study was conducted in Public Hospitals of Central, North West and Western zone of Tigray. Tigray is one region of Ethiopia which covers an area of 109 km^2^ and its elevation is 2084 M above sea level [15–25]. Institutional based cross-sectional study was conducted to assess the knowledge, attitude, and practice towards blood donation and its associated factors among health professionals in Public Hospitals of Central, North West and Western zone of Tigray from August to September 2016.

#### Source and study population

All health professionals who are working in the selected general hospitals in the three zone of Tigray Regional State were the source population. The study populations were randomly selected health professionals who were employees of the selected public hospitals during the data collection time.

#### Sample size determination

The sample size was determined using a single population proportion formula by taking the prevalence rate of practice (32.6%) from Addis Ababa study [11], which gives the largest sample size. Using 95% CI and 5% margin of error, the sample size was found to be 556 after considering 10% non-response rate and design effect of 1.5.

#### Sampling techniques

A multi-stage sampling technique was used to recruit the study participants. At first stage, five general hospitals were selected from total of seven general hospitals in the 3 zones by lottery method. At second stage, random sampling technique was used to select health professionals from human resource list registration as a sampling frame with proportional allocation to the size of each general hospital.

#### Data collection

Data were collected by using a structured and pre-tested self-administered questionnaire.

#### Operational definitions

Based on the mean score, those who score mean and above for knowledge questions were categorized as knowledgeable. In addition, those who score mean and above for attitude question were labeled as having favorable attitude. Regarding blood donation practice, having at least one history of blood donation was used to label them as having practice.

#### Data processing and analysis

All the questionnaires were checked visually, coded, and entered into EPI info version 7.0, then transferred to SPSS version 20 for analysis. Frequencies and proportions were used for descriptive analysis. Binary logistic regression analysis was used to examine association between the explanatory variables and dependant variables. All variables with p value < 0.2 in bivariate logistic regression analysis were entered into the final multiple logistic regression model to identify variables independently associated with KAP towards blood donation. Backward stepwise likelihood ratio was used to select the final independent predictors. The significance of odds ratio (OR) was declared at p value < 0.05.

### Result

#### Socio demographic and factors related with blood donation

A total of 556 health professionals were participated in this study making response rate of 100%. The mean age of study participants were 27.3 ± S.D: 7.5. Majority 218 (39.2%) of the participants were within the age category of 23–29 years and 303 (54.5%) were female. Regarding to marital status and educational level, 293 (52.7%) and 270 (48.6%) were married and degree holders respectively. Of the 556 study participants, 522 (93.8%) were orthodox; 295 (53.1%) had work experience of less than 6 years; 147 (26.4) were served on outpatient department; 313 (56.3%) were with nursing profession. The monthly salary of 277 (49.8%) participant were 1000–3000 ETB and 446 (80.2%) knew their HIV test status (Table 1).

**Table 1:** Socio demographic and factors related with blood donation of health professionals in Tigray public hospitals north Ethiopia August–September 2016 (n = 556)

Variables	Frequency (n = 556)	Percentage (%)
Age		
< 22	35	6.3
23–29	218	39.2
30–35	113	20.3
> 36	190	34.2
Sex		
Male	253	45.5
Female	303	54.5
Marital status		
Married	293	52.7
Single	218	39.2
Divorced	33	5.9
Widowed	12	2.2
Educational status		
Diploma	253	45.5
Degree	270	48.6
Masters and above	20	3.6
Specialist	9	1.6
Others	4	0.7
Religion		
Orthodox	522	93.8
Muslim	27	4.9
Others	7	1.3
Service year (years)		
0–6	295	53.1
7–12	66	11.9
≥ 13	195	35.1
Department		
Inpatient	122	21.9
Operation room	60	10.8
Laboratory	50	9
Out patient	147	26.4
MCH	80	14.4
Pharmacy	42	7.6
Radiology	17	3.1
Others	38	6.8
Profession		
Physician	30	5.4
Nurse	313	56.3
Laboratory	55	9.9
Pharmacy	34	6.1
Midwifery	41	7.4
Others	83	14.9
Monthly income		
1000–3000	277	49.8
3001–6000	244	43.9
6001–9000	24	4.3
> 9000	11	2
HIV test		
Yes	446	80.2
No	110	19.8
Motivation to donate blood		
Yes	421	75.7
No	135	24.3
Knowledge		
Yes	318	57.2
No	238	42.8
Attitude		
Yes	243	43.7
No	313	56.3

Study participants with adequate knowledge on blood donation was 318 (57.2%) whereas 238 (42.8%) were with inadequate knowledge. From the total participants 243 (43.7%) have adequate attitude towards blood donation whereas 313 (56.3%) have inadequate attitude variables (Table 1).

#### Blood donation practice and associated factors among health professionals

Regarding practice, from all participants 266 (47.8%) have ever donated blood at least once in their life time but 290 (52.2%) have never donate. Variables that are significantly associated in bivariable logistic regression analysis only with practice of blood donation were, service year, being motivated by another person to donate and had HIV test previously. In the multi variable logistic regression analysis age greater than 30 years, married health professionals, level of education, adequate knowledge and adequate attitude were found to be significantly associated with practice of blood donation. Those health professionals greater than 30 years old were 2.7 times more likely participated on blood donation practice than those aged ≤ 22 years old (AOR = 2.756 95% CI 1.055–7.197). Married health professionals were 1.7 times more participated on blood donation practice than those single health professionals (AOR = 1.729 95% CI 1.091–2.739). Health professionals having degree in their education level were 69% less likely to participate on blood donation compared to the specialist (AOR = 0.315 95% CI 0.104–0.950).

Participants having adequate knowledge on blood donation were 3.4 times more likely donate blood as compared to inadequate knowledge (AOR = 3.403 95% CI 2.296–5.044). Health professionals having adequate attitude towards blood donation were 3.4 times more likely donated blood than the counter parts (AOR = 3.41 95% CI 2.320–5.041) (Table 2).

**Table 2:** Multivariable logistic regression analysis for factors associated with practice of blood donation among health professionals in Tigray public hospitals, north Ethiopia August–September 2016 (n = 556)

Explanatory variables	Ever donated blood		COR (95% Cl)	AOR (95% Cl)
	Yes	No		
Age				
≤ 22	9 (3.4%)	26 (9%)	1	
23–29	86 (32.3%)	132 (45.5%	1.882 (0.841, 4.211)	1.847 (0.750, 4.547)
≥ 30	171 (64.3%	132 (45.5%	3.742 (1.696, 8.257)	2.756 (1.055, 7.197)*
Marital status				
Single	78 (29.3%)	140 (48.3%	1	
Married	159 (59.8%	134 (46.2%	2.130 (1.486, 3.052)	1.729 (1.091, 2.739)*
Divorced	20 (7.5%)	13 (4.5%)	2.761 (1.303, 5.853)	1.644 (0.679, 3.985)
Widowed	9 (3.4%)	3 (1%)	5.385 (1.416, 20.47)	2.402 (0.549, 10.505)
Educational status				
Specialist	2 (0.8%)	7 (2.4%)	1	
Diploma	132 (49.6%	121 (41.7%	1.284 (0.911, 1.811)	0.950 (0.626, 1.443)
Degree	124 (46.6%	146 (50.3%	0.505 (0.188, 1.352)	0.315 (0.104, 0.95)*
Masters and above	6 (2.3%)	14 (4.8%)	0.336 (0.069, 1.649)	0.370 (0.070, 1.954)
Others	2 (0.7%)	2 (0.8%)	1.177 (0.163, 8.481)	1.127 (0.116, 10.926)
Service year (years)				
0–6	115 (43.2%	180 (62.1%	1	
7–12	29 (10.9%)	37 (12.8%)	1.227 (0.715, 2.104)	1.124 (0.575, 2.198)
≥ 13	122 (45.9%	73 (25.1%)	2.616 (1.802, 3.797)	1.538 (0.904, 2.619)
Motivation donate blood				
No	54 (20.3%)	81 (27.9%)	1	
Yes	212 (79.7%	209 (72.1%	1.522 (1.026, 2.256)	0.623 (0.387, 1.003)
HIV test in the past				
No	225 (84.6%	221 (76.2%	1	
Yes	41 (15.4%)	69 (23.8%)	0.58 (0.380, 0.896)	0.665 (0.398, 1.110)
Knowledge				
In adequate	73 (27.4%)	165 (56.9%	1	
Adequate	193 (72.6%	125 (43.1%	3.490 (2.455, 4.982)	3.403 (2.296, 5.044)*
Attitude				
In adequate	108 (40.6%	205 (70.7%	1	
Adequate	158 (59.4%	85 (29.3%)	3.528 (2.482, 5.016)	3.419 (2.320, 5.041)*

*p value ≤ 0.05 considered as statistical significant

## Discussion

This study has assessed blood donation practice and associated factors among health professionals in Tigray regional state public hospitals. Nearly half of the study participants 47.8% reported that they have ever donated blood at least once in their life time. This finding is higher than the finding of the study conducted in University of Gondar Hospital; Northwest Ethiopia which reported that blood donation practice among health professionals was 33.2% [6]. A study in Debre Markos town, northwest Ethiopia also reported that 32.6% of health professionals have ever donated blood at least once in their life time [8]. It is also higher than similar studies conducted in Addis Ababa among health professionals of Tukur Anbessa hospital 38.3% [7]. This difference could be due to repeated campaigns conducted in our study area by the regional health bureau and other blood bank centers in the region.

Age of health professionals 30 years or above was positively associated with blood donation practice. Those age 30 years or above were 2.76 times more likely to donate blood than those with age 22 years or below. Similar findings were also reported in other part of Ethiopia [6]. The probable reason may be as their age increases their awareness to donate blood through time increases.

Married health professionals were 1.7 times more likely to donate than those who were single. This is contradicting with a study from Lagos Nigeria which reported willingness to donate blood was positively associated with being single was a significant predictor of blood donation [5]. This difference may be due to socio-demographic difference of the two populations.

In this study having higher level of formal education is significantly associated with blood donation practice. This is supported by the study from Debre Markos town, northwest Ethiopia which revealed that health professionals who attended certificate and above education were more likely to donate blood [5]. This may be due to the fact that having higher education is associated with exposing to frequent blood donation campaigns and blood donation related information to have better knowledge, attitude and then better blood donation practice.

Having adequate knowledge about blood donation is positively associated with blood donation practice. Health professionals who had adequate knowledge of blood donation were 3.4 times more likely to donate blood than those who had inadequate knowledge. This is supported by a study from Debre Markos, Northwest Ethiopia which reported that the practice of blood donation was higher among respondents who were knowledgeable [8]. Another study conducted in Gondar Hospital, Northwest Ethiopia also revealed that being knowledgeable is associated with blood donation practice [6]. That is also supported by studies done in Addis Ababa health facility health care workers in Ethiopia [7]. This justifies that having more knowledge about importance of blood donation makes health professionals to donate blood.

Health professionals having adequate attitude about blood donation were 3.4 times more likely to participate on blood donation than those having inadequate attitude. This is supported by the study from Debre Markos, Northwest Ethiopia which reported favorable attitude is significantly associated with blood donation practice [8]. The study from Gondar University hospital, Northwest Ethiopia also revealed that having favorable attitude is a predictor to blood donation practice [6]. This is supported by a study done in Addis Ababa health facility [26].

## Conclusion

Nearly half of the health professionals included in the study reported they donated blood at least once in their life time. Age of health professionals 30 years or above, being married, having formal education, having adequate knowledge and having adequate attitude about blood donation were factors associated significantly with blood donation practice among health professionals in public health institutions of Tigray regional state in Ethiopia. So that, the Red Cross and Red Crescent Associations together with respective government ministries need to engage on promoting blood donation by designing different strategies that can improve knowledge and attitude of people about blood donation. The blood bank offices at different levels also need to conduct repeated blood donation campaigns at health institutions to encourage blood donation by health professionals.

## Limitation

This study was conducted through cross-sectional study and may not show the cause and effect relationship.

## Abbreviations

CI: confidence interval
AOR: adjusted odd ratio
SPSS: Statistical Package for Social Sciences
